# Anatomical Mechanisms Underlying Clinically Reported Complications of the Infraclavicular Brachial Plexus Block: A Narrative Review

**DOI:** 10.3390/jcm15051931

**Published:** 2026-03-03

**Authors:** Petar-Preslav Petrov, Delyan Dimitrov, Darina Barbutska, Rumyana Etova

**Affiliations:** 1 Department of Anatomy, Histology and Embriology, Medical University of Plovdiv, 4002 Plovdiv, Bulgaria; 2 Faculty of Medicine, Medical University of Plovdiv, 4002 Plovdiv, Bulgaria; 3 Department of Epidemiology and Disaster Medicine, Section of Disaster Medicine, Medical University of Plovdiv, 4002 Plovdiv, Bulgaria

**Keywords:** infraclavicular block, brachial plexus, peripheral nerve block, upper limb anesthesia, regional anesthesia, infraclavicular region, complications

## Abstract

**Background**: The infraclavicular brachial plexus block is a widely used regional anesthesia technique for surgery of the distal upper limb. Although generally considered safe—particularly with ultrasound guidance—a range of vascular, neurological, respiratory, and anesthetic-related complications continues to be reported. Understanding how anatomic factors can influence the occurrence of these events is essential for improving procedural safety. **Objective**: This narrative review aims to correlate clinically reported complications of the infraclavicular block with underlying anatomical mechanisms that may predispose to their development. **Methods**: A narrative review of the literature was conducted using PubMed, Scopus and Web of Science to identify clinical studies, observational series, and case reports published between 1995 and 2025 that documented complications associated with infraclavicular brachial plexus block in adults. Publications were selected based on relevance to vascular, neurological, respiratory, infectious, and local anesthetic systemic complications. Findings were synthesized descriptively, with emphasis on anatomical-clinical correlations rather than quantitative meta-analysis. **Results**: Reported complications include vascular puncture and hematoma formation, transient or persistent neurological deficits, Horner’s syndrome, hemidiaphragmatic paralysis, pneumothorax, local anesthetic systemic toxicity, and infectious complications. The incidence of these events varies widely across studies, reflecting differences in block technique, use of ultrasound guidance, injected anesthetic volume, and operator experience. Anatomical factors—such as the close relationship of the cords of the brachial plexus to the axillary vessels and the continuity of fascial planes—provide plausible explanations for these variations. **Conclusions**: Most complications of the infraclavicular block can be understood and anticipated through careful consideration of regional anatomy. Integrating anatomical knowledge with ultrasound guidance and optimized injection strategies may substantially reduce the risk of adverse events. This review highlights key anatomical mechanisms underlying reported complications and outlines practical implications for clinical practice.

## 1. Introduction

In recent years, regional anesthesia has become an integral component of modern anesthesiology practice, providing effective perioperative analgesia while avoiding many of the systemic effects associated with general anesthesia [1]. Among peripheral nerve block techniques, brachial plexus blocks are widely used for surgical procedures of the upper limb. The infraclavicular block is specifically designed to anesthetize the cords of the brachial plexus and is commonly employed for procedures involving the elbow, forearm, and hand [2,3].

The widespread adoption of ultrasound guidance has further improved block success rates and contributed to a reduction in complication rates. Nevertheless, clinically significant adverse events continue to be reported, ranging from vascular puncture and neurological injury to respiratory impairment and local anesthetic systemic toxicity [4].

Many of these complications are not random but can be explained by the complex anatomy of the infraclavicular region. The cords of the brachial plexus are in close proximity to the axillary artery and vein and are enclosed within a complex tissue plane that permits local anesthetic spread beyond the intended target area [5]. Variations in individual anatomy, needle trajectory, and the volume of injected local anesthetic may influence both block efficacy and the likelihood of complications.

Previous publications addressing complications of infraclavicular brachial plexus block have often focused on complication rates or technical comparisons between different approaches. A clearer understanding of how regional anatomy contributes to the development of adverse events may help clinicians anticipate potential risks and refine their block techniques.

The aim of this narrative review is to synthesize clinically reported complications of infraclavicular brachial plexus block and to correlate them with the underlying anatomical mechanisms that predispose to their occurrence.

## 2. Materials and Methods

This article was designed as a narrative review. A structured literature search was conducted in PubMed, Scopus, and Web of Science to identify publications addressing complications associated with infraclavicular brachial plexus block. The search covered the period from January 1995 to January 2025.

The search strategy included combinations of the following keywords: (“infraclavicular block” OR “infraclavicular approach” OR “infraclavicular brachial plexus” OR “infraclavicular brachial plexus block” OR “infraclavicular brachial plexus blockade” OR “infraclavicular nerve block” OR “infraclavicular ultrasound-guided brachial plexus block”) AND (complication* OR “vascular puncture” OR “neurological injur*” OR “neurological complication*” OR pneumothorax OR infection OR “local anesthetic systemic toxicity” OR “Horner* syndrome” OR “diaphragmatic paralysis” OR “hemidiaphragmatic paralysis”).

The initial search yielded 1040 records (297 in PubMed, 390 in Scopus, and 353 in Web of Science). After removal of duplicates, 586 unique records remained for screening.

Titles and abstracts were screened for relevance, followed by full-text evaluation of potentially eligible publications. Of these, 24 studies met the inclusion criteria and were included in the qualitative synthesis. Two additional relevant publications were identified through manual screening of reference lists, resulting in a total of 26 included studies.

Clinical studies, observational cohorts, randomized and non-randomized trials, and case reports involving adult patients were considered eligible if they reported one or more complications related to the infraclavicular approach. Animal studies, purely technical descriptions without clinical outcomes, and publications not addressing complications were excluded, except where relevant to safety considerations or low complication rates.

Data were synthesized qualitatively, with emphasis on identifying recurrent anatomical and technical factors contributing to reported complications rather than on quantitative pooling of results.

## 3. Results and Discussion

### 3.1. Anatomy of the Infraclavicular Region

Safe performance of the infraclavicular brachial plexus block requires a detailed understanding of the three-dimensional anatomy of the infraclavicular region and the spatial relationships within the neurovascular bundle. At this level, the brachial plexus is encountered as three cords, which constitute the principal anatomical target of the infraclavicular approach.

Topographically, the infraclavicular region lies inferior to the clavicle and medial to the humerus. It is bounded anteriorly by the pectoralis major and minor muscles, medially by the thoracic wall, superiorly by the clavicle and coracoid process, and laterally by the proximal humerus. Immediately deep to the pectoral muscles lies the neurovascular bundle, which contains the cords of the brachial plexus as well as the axillary artery and vein [4]. The close anatomical relationship among these structures underlies both the effectiveness of the block and the potential for vascular complications.

The brachial plexus is formed by the anterior rami of the C5–T1 spinal nerves, with occasional contributions from C4 and/or T2. As the plexus passes beneath the clavicle, it transitions from the supraclavicular to the infraclavicular region, where its components reorganize into three cords—lateral, medial, and posterior—named according to their position relative to the axillary artery. At this level, the cords typically surround the second part of the axillary artery, and their intimate relationship with the axillary vessels is of critical clinical relevance [6,7,8,9,10,11,12].

Magnetic resonance imaging studies of the infraclavicular region have demonstrated that the brachial plexus is typically located directly beneath the clavicle in a parasagittal plane approximately 1 cm medial to the coracoid process. These findings suggest that plexus depth may be reasonably estimated using fixed bony landmarks, thereby supporting the concept of anatomical “depth gauging” prior to needle advancement. Importantly, in this same parasagittal plane, the lung is rarely encountered and, when present, is generally positioned posterior to the plexus [13]. This spatial relationship has direct implications for pleural safety and helps to explain the relatively low incidence of pneumothorax when needle trajectory is carefully controlled.

Traditional anatomical descriptions have referred to a fascial “sheath” enclosing the brachial plexus as it extends from the supraclavicular region into the axilla. However, this concept has been critically re-examined. Anatomical analyses have questioned the existence of a discrete, closed fascial compartment surrounding the plexus [14]. Rather than being separated by a well-defined anatomical barrier, the cords of the brachial plexus and the axillary vessels coexist within a shared connective tissue environment.

From a mechanistic perspective, local anesthetic spread within this region is unlikely to represent simple filling of a closed compartment governed solely by bulk hydrostatic flow. Instead, emerging experimental evidence suggests that transport along and across tissue planes may exhibit directionality and structural anisotropy, influenced by collagen fiber orientation, septal architecture, and differences in tissue compliance. Recent evidence further indicates that the distribution of local anesthetic may also involve diffusion-compatible interstitial transport mechanisms that are not fully captured by dye- or contrast-based models, which primarily demonstrate macroscopic fluid displacement [15]. Accordingly, spread within the infraclavicular region should be understood as a composite process involving constrained flow, structural channeling, and physicochemical transport within a dynamically compliant connective tissue network.

This refined interpretation moves beyond the notion of a static “sheath” and supports the concept of a structurally guided, but not completely enclosed, neurovascular tissue plane in which the extent and direction of anesthetic spread depend on both anatomical architecture and injection-related factors.

This configuration has been described as the “axillary tunnel”, a space bounded by relatively rigid anatomical structures—including the clavicle, coracoid process, thoracic wall, and surrounding musculature—which together constrain the neurovascular bundle [16]. Within this framework, the neurovascular structures are situated in a complex connective tissue plane rather than within an isolated fascial sheath. The contours of the rigid boundaries and the limited compliance of the surrounding tissues influence the direction and extent of injectate spread.

Importantly, the cords and the axillary vessels are not separated by a distinct anatomical septum; instead, they lie within the same tissue plane. This anatomical arrangement may explain both the effectiveness of circumferential local anesthetic spread around the artery and the risk of inadvertent intravascular injection. The dynamics of local anesthetic distribution appear to be governed not by containment within a closed compartment, but by the continuity of tissue planes and the geometric constraints imposed by adjacent structures [5]. When injectate volume increases within this confined space, the resulting pressure gradients may direct its spread along the path of least resistance within the connective tissue planes.

The axillary artery, the principal arterial supply to the upper limb, extends from the subclavian artery at the lateral border of the first rib and continues as the brachial artery at the lower border of the teres major muscle [17]. The axillary vein originates at the lower border of the teres major muscle and continues as the subclavian vein at the outer border of the first rib [11]. Microscopically, the axillary artery is a muscular-type artery characterized by a thick tunica media rich in smooth muscle fibers, whereas the axillary vein is a medium-sized vein with a thinner, collagen-rich tunica media [18,19,20,21]. These structural differences may influence the clinical consequences of inadvertent vascular puncture.

**Figure 1:**
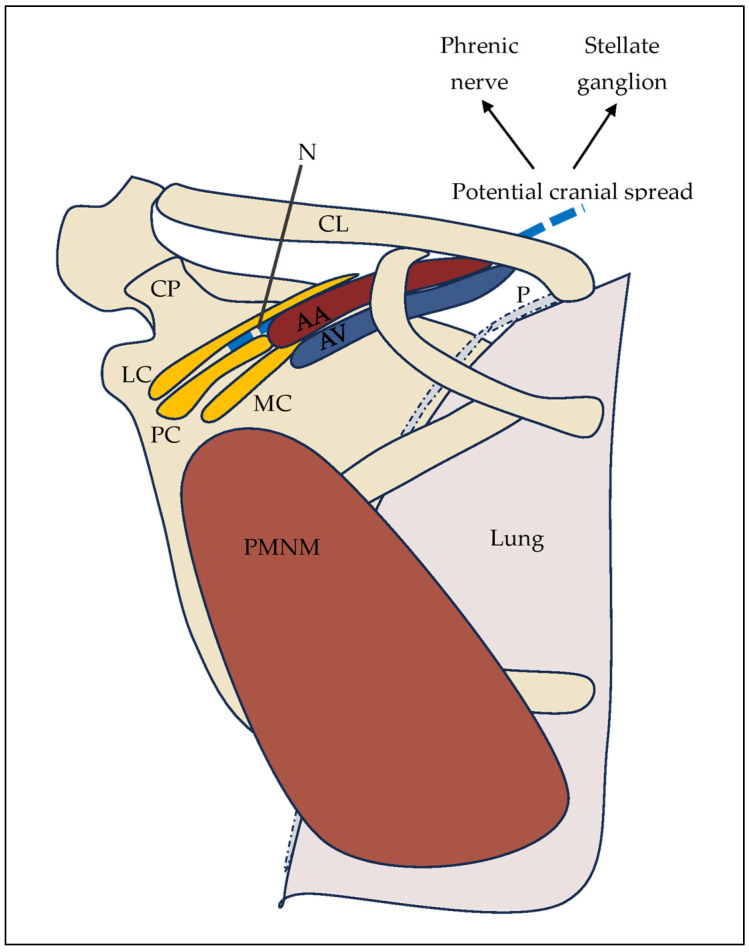
Anatomical relationships in the infraclavicular region demonstrating the spatial proximity of the brachial plexus cords to the axillary vessels, pleura, and phrenic nerve pathways, illustrating potential mechanisms underlying vascular puncture and cranial spread of local anesthetic. Abbreviations: CL—clavicle; AA—axillary artery; AV—axillary vein; LC—lateral cord; PC—posterior cord; MC—medial cord; PMNM—pectoralis minor muscle; CP—coracoid process.

Taken together, the infraclavicular region should be conceptualized not as a closed fascial compartment but as a constrained neurovascular space defined by tissue plane continuity and rigid anatomical boundaries. This perspective provides a coherent anatomical explanation for both the success and the variability of infraclavicular block techniques (Figure 1).

### 3.2. Infraclavicular Block—Definition, Anatomical Considerations, and Technical Aspects

The infraclavicular block is a regional anesthesia technique that targets the cords of the brachial plexus and represents one of the principal approaches to peripheral nerve blockade of the upper limb. It was developed in part to reduce the risk of pneumothorax, which is more commonly associated with the supraclavicular approach. Clinically, the infraclavicular block is primarily used to provide anesthesia and analgesia for surgical procedures involving the elbow, forearm, and hand [8,22,23,24].

The block does not reliably anesthetize the shoulder region, as shoulder innervation is predominantly provided by the supraclavicular nerves and the axillary nerve, which branch proximally and are not consistently affected at the infraclavicular level. Similarly, cutaneous innervation of the upper arm may be incomplete due to the lack of blockade of the intercostobrachial nerve and intercostal nerves. These anatomical considerations are important when interpreting cases of incomplete block or unexpectedly preserved sensation in clinical practice.

Historically, the infraclavicular approach was first described in the early twentieth century and was subsequently refined with the introduction of nerve stimulation and, later, ultrasound guidance. The ultrasound-guided infraclavicular block represents a major advancement, enabling direct visualization of the axillary vessels and the cords of the brachial plexus, thereby improving block success rates and influencing the incidence and pattern of reported complications [25,26,27,28,29].

**Figure 2:**
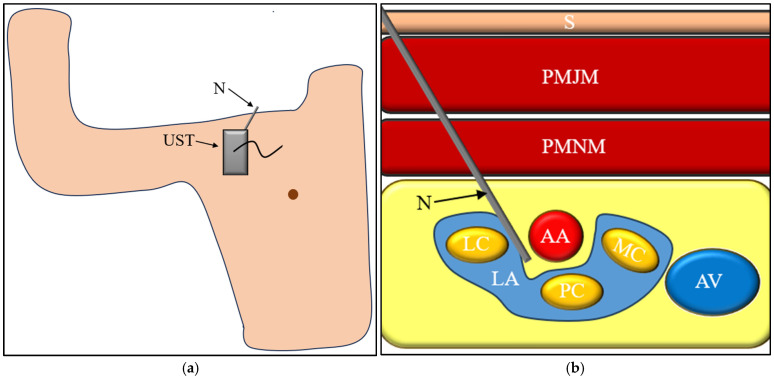
Ultrasound-Guided Infraclavicular Brachial Plexus Block Technique. (**a**) Patient positioning for ultrasound-guided infraclavicular brachial plexus block. The upper limb is positioned in less than 90° of shoulder abduction and less than 90° of elbow flexion to optimize access to the infraclavicular region. The ultrasound transducer is oriented horizontally in the parasagittal plane inferior to the clavicle. Abbreviations: N—needle, UST—ultrasound transducer. (**b**) Corresponding ultrasound cross-sectional view of the infraclavicular region demonstrating the layered anatomy, including skin (S) and subcutaneous tissue, pectoralis major muscle (PMJM), pectoralis minor muscle (PMNM), the advancing needle (N), axillary artery (AA), axillary vein (AV), and the three cords of the brachial plexus—lateral (LC), posterior (PC) and medial cord (MC). Local anesthetic (LA) spread surrounding the neurovascular structures is illustrated.

From a technical perspective, several factors influence both the effectiveness and safety of the block, including needle trajectory, proximity to the axillary vessels, and the volume of injected local anesthetic. Contemporary ultrasound-guided techniques typically involve circumferential (U-shaped) deposition of local anesthetic around the axillary artery to facilitate spread within the tissue planes of the neurovascular bundle. Injection volumes generally range from 20 to 30 mL; however, larger volumes have been associated with an increased risk of unpredictable spread and systemic toxicity. These technical variables are particularly relevant when analyzing the mechanisms underlying vascular, neurological, respiratory, and anesthetic-related complications [30,31,32] (Figure 2).

### 3.3. Complications of the Infraclavicular Block

**Figure 3:**
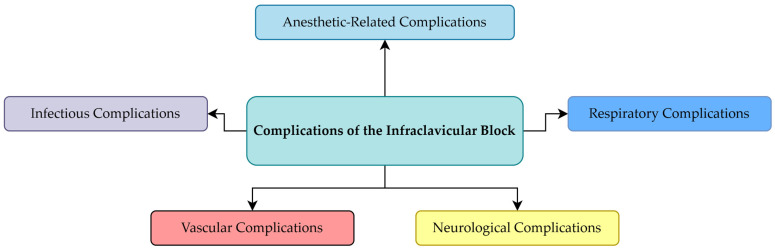
Classification of Clinically Reported Complications of the Infraclavicular Brachial Plexus Block.

The following sections address the principal categories of complications reported after infraclavicular brachial plexus block, with particular emphasis on their anatomical basis and clinical relevance: (Figure 3).

1. Vascular complications
  o Puncture of the axillary artery or vein (with or without hematoma formation)
  o Inadvertent intravascular injection of local anesthetic
2. Neurological complications
  o Dysesthesia and paresthesia
  o Horner’s syndrome
  o Diaphragmatic paralysis
3. Respiratory complications
  o Pneumothorax
4. Anesthetic-related complications
  o Local anesthetic systemic toxicity (LAST)
5. Infectious complications
  o Infection at the site of anesthetic administration

The reported incidence and profile of complications vary according to the technical approach used for infraclavicular brachial plexus block. Landmark-based and vertical techniques, nerve stimulator-guided approaches, and ultrasound-guided methods differ in their ability to visualize vascular structures, neural elements, and needle trajectory. Consequently, the type and frequency of reported complications appear to be influenced not only by anatomical factors, but also by the degree of real-time visualization and control during needle advancement and local anesthetic injection.

Although direct comparison across studies is limited by heterogeneity in study design, patient populations, and injected volumes, available clinical reports allow for a qualitative comparison of complication patterns associated with different techniques. The following table summarizes the most commonly reported complications and their relative frequency across approaches, based on published clinical data (Table 1).

The available literature on infraclavicular block complications includes randomized trials and cohort studies but also numerous small observational reports and case descriptions. Accordingly, the evidentiary strength varies, and incidence estimates are primarily derived from larger comparative datasets.

#### 3.3.1. Vascular Puncture

In the infraclavicular region, the axillary artery and vein lie in close proximity to the cords of the brachial plexus, rendering them susceptible to injury during infraclavicular brachial plexus block. This anatomical relationship represents the primary mechanism underlying vascular puncture, even when contemporary techniques are employed.

**Figure 4:**
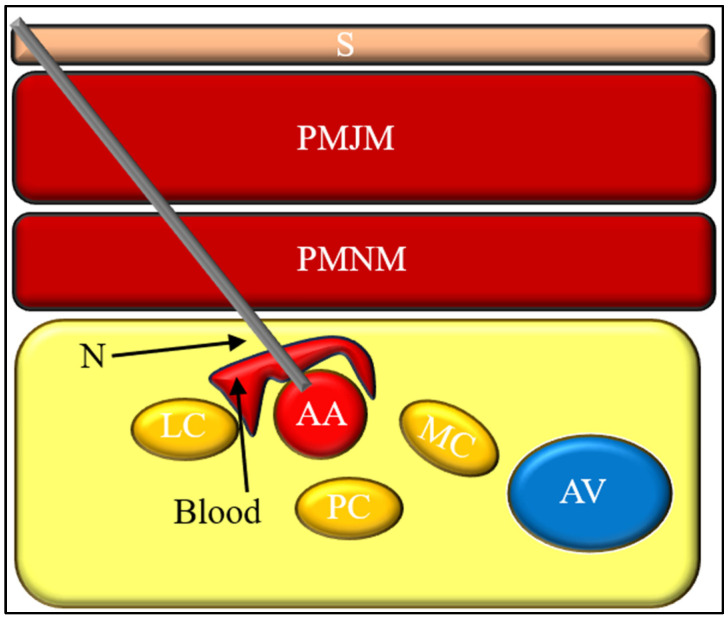
Mechanism of vascular puncture during infraclavicular brachial plexus block. Inadvertent needle advancement into the axillary artery (AA) may result in intravascular injection or hematoma formation. The close anatomical relationship between the axillary vessels and the cords of the brachial plexus (LC, PC, MC) underscores the importance of continuous needle tip visualization.

Vascular puncture during infraclavicular block may result in bleeding and hematoma formation within the infraclavicular region (Figure 4). Hematomas may range from superficial and clinically insignificant to deep collections capable of compressing adjacent neural structures, potentially leading to secondary neurological symptoms. The risk of clinically significant hematoma formation is increased in patients with coagulation disorders or in those receiving anticoagulant therapy [45,46].

Incidents of vascular puncture during infraclavicular block have been described in numerous clinical studies. For example, as early as 1995, Kilka et al. reported vascular puncture in 18 of 175 patients (10%) undergoing a vertical infraclavicular approach, with all punctures involving the axillary vein and no arterial or pleural injury was reported [33]. Jandard et al. reported a 5% incidence of vascular puncture in patients undergoing a paracoracoid approach using nerve stimulator guidance [34]. Rodríguez et al. documented vascular complications or hematoma formation in 5% of patients when comparing single- and double-injection techniques [47]. Koscielniak-Nielsen reported a lower incidence of vascular puncture (2%) with infraclavicular block compared with the supraclavicular approach [48].

Cases of clinically significant hematoma have also been reported. Gleeton et al. described a symptomatic axillary fossa hematoma following ultrasound-guided infraclavicular block in a patient with an undiagnosed mycotic aneurysm, highlighting the role of underlying vascular pathology [49]. Additional studies have reported vascular puncture rates ranging from 3% to 7%, depending on the technique and patient population [41,50,51,52].

Vascular puncture and local anesthetic systemic toxicity have been reported even in experienced hands. A single randomized study suggested a potential reduction in arterial puncture with hydrodissection; however, these findings originate from a limited sample and require confirmation in larger comparative trials. In a randomized study, Er et al. demonstrated that arterial puncture occurred only in patients who underwent infraclavicular block without hydrodissection, whereas no vascular punctures were observed when hydrodissection was used [35]. Similar findings regarding the protective effect of hydrodissection have been reported in peripheral nerve blocks [53].

Prevention of vascular complications relies primarily on meticulous technique and real-time ultrasound guidance. Ultrasound allows visualization of the brachial plexus cords, axillary vessels, needle trajectory, and spread of the injectate, thereby reducing—but not eliminating—the risk of vascular puncture [54,55]. Aspiration before injection remains essential for early detection of inadvertent intravascular needle placement. The axillary vein, although more superficial and compressible, lies medial to the axillary artery, while the cords of the brachial plexus directly surround the artery. Despite the deeper location of the artery, this anatomical configuration may predispose to arterial puncture [17,56].

Control of arterial bleeding in the infraclavicular region may be challenging because of the deep anatomical location of the axillary artery beneath the pectoralis major and minor muscles and its proximity to the coracoid process. The overlying muscular layers limit the ability to apply effective external compression against a firm posterior structure, particularly given the absence of a readily compressible surface comparable to more superficial arterial sites. Furthermore, the relatively high intraluminal pressure within the axillary artery, compared with the adjacent vein, may contribute to more persistent bleeding in the event of arterial puncture. In such cases, prolonged firm compression is recommended, and careful clinical observation is required. If bleeding persists or expanding hematoma is suspected, prompt surgical or vascular consultation may be necessary.

#### 3.3.2. Neurological Complications

##### Nerve Injury

It is important to distinguish transient sensory symptoms from confirmed structural nerve injury, as many reported events represent temporary phenomena rather than true permanent deficits.

Neurological complications following infraclavicular brachial plexus block are rare and are most often transient. While some authors, such as Liguori, report transient neurological symptoms in up to 10–15% of patients after regional anesthesia, persistent nerve injury remains exceedingly rare [57].

Peripheral nerve injury may occur as a result of direct mechanical trauma from the needle, intraneural injection, ischemic or chemical injury. Anatomically, peripheral nerves are protected by three layers of connective tissue—the epineurium, perineurium, and endoneurium—with the perineurium playing a key role in maintaining the blood–nerve barrier [58,59,60]. Disruption of the perineurium increases nerve vulnerability and may lead to clinically significant neurological deficits.

Despite the use of ultrasound guidance, inadvertent nerve puncture may still occur during infraclavicular block. The occurrence of paresthesia during needle insertion or injection is considered a clinical marker of possible intraneural needle placement and is associated with an increased risk of postoperative neurological symptoms [61,62,63].

Large clinical studies demonstrate a low incidence of neurological complications associated with infraclavicular block. For example, Keschner et al. reported no neurological complications among 248 patients [64]. Neurological symptoms following infraclavicular block were reported by Fredrickson et al. in 5 of 30 patients (17%) [65]. Koscielniak-Nielsen et al. documented 8 cases of paresthesia and/or pain during injection [48]. Fredrickson and Kilfoyle reported neurological symptoms in 9 of 122 patients, with only one case directly attributed to the block (7%) [66].

The use of ultrasound further reduces the incidence of clinically significant nerve injury. In a cohort of 627 patients, Lecours et al. documented only 4 cases of neurological symptoms following infraclavicular block, all of which were transient [38]. Similar results were reported by Vazin et al., who described late-onset dysesthesia in 13% of patients, without evidence of permanent nerve injury [67].

Overall, available clinical data suggest that most neurological symptoms are transient and rarely result in permanent deficits, particularly when ultrasound guidance and meticulous technique are employed.

##### Horner’s Syndrome

Horner’s syndrome is a neurological complication characterized by ptosis, miosis, and anhidrosis on the affected side of the face. Its occurrence during brachial plexus blockade is attributed to concomitant blockade of sympathetic fibers supplying the eye and face through involvement of the stellate ganglion [68,69].

Although Horner’s syndrome is more commonly associated with interscalene and supraclavicular blocks, it may also be observed following infraclavicular block, particularly when large volumes of local anesthetic are used or during continuous infusions [70,71,72]. The proposed mechanism involves medial and cranial spread of local anesthetic from the infraclavicular region toward the cervical sympathetic chain.

Anatomically, the stellate ganglion—formed by the fusion of the inferior cervical (C7) and first thoracic (T1) sympathetic ganglia—is located in close proximity to the brachial plexus and the subclavian artery. Direct in vivo confirmation of cranial spread from the infraclavicular region remains limited, and the proposed mechanism is largely inferential [73].

Clinical reports indicate that Horner’s syndrome following infraclavicular block is usually transient and does not result in long-term sequelae. For example, Salengros et al. reported a case following continuous infusion in which symptoms resolved rapidly after discontinuation of the block [74]. Similar observations were reported by Walid et al. after the use of moderate volumes of local anesthetic [75]. Rodríguez et al. noted an increased incidence of Horner’s syndrome (3%) with double-injection techniques, suggesting a dependence on both volume and technique [47].

The literature reports variable incidences of Horner’s syndrome among different block techniques, generally ranging between 4% and 12%, with higher rates observed in interscalene and supraclavicular blocks [33,34,39,50]. Anatomical studies suggest that continuity of tissue planes may influence the direction and extent of local anesthetic spread [76,77,78].

Based on available reports, Horner’s syndrome following infraclavicular block appears to be an infrequent and typically transient finding. The occurrence of this syndrome may be reduced by limiting the volume of local anesthetic and carefully planning the injection technique.

##### Diaphragmatic Paralysis

Diaphragmatic paralysis is a clinically significant but relatively rare complication following infraclavicular brachial plexus block. It results from inadvertent blockade of the phrenic nerve, leading to ipsilateral hemidiaphragmatic paralysis. Although this complication is much more frequently associated with interscalene and supraclavicular approaches, cases of diaphragmatic paralysis have also been reported following infraclavicular block [79,80].

The primary proposed mechanism is cranial spread of local anesthetic from the infraclavicular space toward the cervical region, facilitated by the continuity of tissue planes. This cranial spread allows the local anesthetic to reach the phrenic nerve, which originates from the C3–C5 nerve roots and courses in close proximity to the subclavian vessels before entering the thoracic cavity [81].

The incidence of hemidiaphragmatic paralysis varies widely depending on the block approach, the volume of local anesthetic used, and the method of assessment. Interscalene block is associated with the highest incidence, followed by supraclavicular techniques, with reported rates of up to 70% [82,83,84]. In contrast, infraclavicular block demonstrates a lower but non-negligible incidence, with reported values ranging approximately from 3% to 24% [41,85].

Clinically significant respiratory compromise is rare but has been described, particularly in patients with limited pulmonary reserve. Gentili et al. [86] reported a case of acute respiratory failure in an elderly patient with chronic obstructive pulmonary disease following infraclavicular block, characterized by hypercapnia, hypoxemia, ipsilateral diaphragmatic elevation, and lower lobe atelectasis. The patient recovered with supportive treatment, and diaphragmatic function normalized within 24 h [86].

Prospective studies further support the association between infraclavicular block and altered diaphragmatic function. Rettig et al. [39] identified ipsilateral diaphragmatic motion impairment in 26% of patients (9 of 35) following vertical infraclavicular block using relatively large volumes of ropivacaine. The authors noted a correlation between the presence of Horner’s syndrome and diaphragmatic dysfunction, suggesting a shared anatomical mechanism related to cranial spread of the local anesthetic [39].

Comparative studies highlight the advantages of the infraclavicular approach over more proximal techniques. Petrar et al. demonstrated a significantly lower incidence of complete hemidiaphragmatic paralysis following infraclavicular block compared with supraclavicular block when identical volumes of local anesthetic were used [40]. Similarly, Parameswari et al. reported a significantly lower incidence of diaphragmatic paralysis with infraclavicular block when smaller volumes of local anesthetic were employed [41].

The available data suggest that diaphragmatic dysfunction may be volume dependent and associated with cranial spread of local anesthetic, although definitive causal relationships cannot be established based on current evidence. Although usually transient and clinically silent in healthy individuals, this complication warrants careful consideration in patients with pre-existing respiratory disease. Strategies such as minimizing anesthetic volume and avoiding unnecessary proximal spread may further reduce the risk.

From a mechanistic perspective, the continuity of tissue planes between the infraclavicular and cervical regions provides a potential pathway for proximal migration of local anesthetic toward the phrenic nerve [5,14,16]. Experimental and anatomical observations suggest that injectate spread within these compartments is influenced by mechanical constraints and directionality rather than by simple bulk flow [70]. In this context, external supraclavicular pressure has been proposed as a maneuver to transiently modify tissue-plane dynamics and potentially limit cranial tracking of local anesthetic. Although robust clinical data are lacking, this concept illustrates how mechanical manipulation of interfascial pathways may influence anesthetic distribution.

While comparative studies demonstrate lower incidence with infraclavicular techniques, mechanistic conclusions regarding cranial spread are largely extrapolated from imaging and functional assessments rather than direct visualization studies.

#### 3.3.3. Pneumothorax

Pneumothorax is a rare but potentially serious complication of infraclavicular brachial plexus block. It results from inadvertent pleural puncture, allowing air to enter the pleural space and leading to partial or complete lung collapse [87,88,89,90,91]. Compared with the supraclavicular approach, infraclavicular block is associated with a significantly lower risk of pneumothorax; however, this risk is not completely eliminated [92].

The primary mechanism involves excessive needle advancement beyond the target depth, as well as medial or posterior redirection of the needle. Although the cords of the brachial plexus are typically located at a depth of approximately 4.5–6 cm, anatomical variations, patient body habitus, and differences among infraclavicular techniques may place the pleura within reach of the needle, particularly with parasagittal or vertical approaches [92]. Magnetic resonance imaging studies have demonstrated that, in the parasagittal plane approximately 1 cm medial to the coracoid process, the lung is rarely encountered directly anterior to the plexus and, when present, is generally located posterior to it [13]. This anatomical relationship suggests that pleural injury is less likely to result from standard needle advancement within the correct plane and depth and is more likely to occur when the needle trajectory deviates medially or posteriorly, or when visualization of the needle tip is lost.

Clinically reported cases demonstrate that pneumothorax may occur despite ultrasound guidance. Neuburger et al. [42] described a severe case following vertical infraclavicular block, complicated by pleural effusion and postoperative pulmonary infection. The authors emphasized that the risk of pneumothorax with the vertical infraclavicular approach ranges from 0.2% to 0.7% [42].

Subsequent case reports have described similar events, illustrating potential failure mechanisms rather than providing reliable incidence estimates. Crews et al. documented a case of pneumothorax following a coracoid infraclavicular block, while Sanchez et al. reported delayed presentation of apical pneumothorax in two young patients undergoing upper limb surgery. Importantly, symptoms developed on the first or second postoperative day, highlighting the potential for delayed clinical manifestation [93,94].

**Figure 5:**
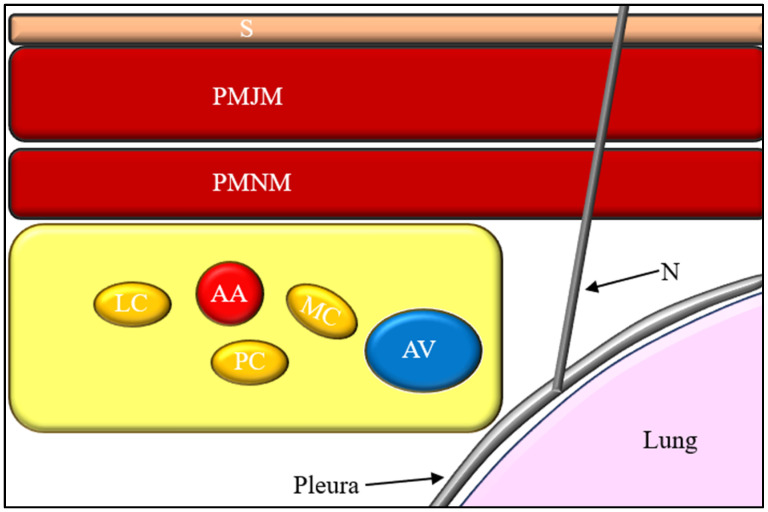
Mechanism of pneumothorax during infraclavicular brachial plexus block. Excessively medial or deep needle advancement may result in pleural puncture, with potential injury to the underlying lung parenchyma. The spatial relationship between the infraclavicular neurovascular bundle and the pleura underscores the importance of controlled needle trajectory and continuous visualization. Abbreviations: S—skin, PMJM—pectoralis major muscle, PMNM—pectoralis minor muscle, AA—axillary artery, AV—axillary vein, LC—lateral cord, PC—posterior cord, MC—medial cord, N—needle.

Large cohort studies confirm the overall low incidence of this complication. In a retrospective analysis of more than 6000 brachial plexus blocks, Gauss et al. [43] reported only four cases of pneumothorax, two of which occurred after infraclavicular block, corresponding to an incidence of approximately 0.07%. Notably, in both cases, the infraclavicular blocks were performed under ultrasound guidance, underscoring the fact that ultrasound reduces but does not completely eliminate the risk [43]. The likely cause of pneumothorax is loss of sight of the needle tip during insertion (Figure 5).

Clinically, pneumothorax may present with dyspnea, pleuritic chest pain, cough, or hypoxemia; however, symptoms may be mild or delayed in onset. This is particularly relevant in ambulatory practice, where patients may be discharged before symptom onset. Therefore, a high index of suspicion and appropriate postoperative instructions are required, especially in patients with underlying pulmonary disease.

In conclusion, pneumothorax following infraclavicular brachial plexus block is a rare complication and occurs significantly less frequently compared with more proximal approaches. Nevertheless, thorough knowledge of needle depth and trajectory, as well as chest wall anatomy, remains critical. Ultrasound guidance, proper needle orientation, and avoidance of excessive medial advancement are key strategies for minimizing this potentially serious complication.

#### 3.3.4. Anesthetic-Related Complications: Local Anesthetic Systemic Toxicity (LAST)

Local anesthetic systemic toxicity (LAST) is a rare but potentially life-threatening complication of infraclavicular brachial plexus block. It primarily affects the central nervous and cardiovascular systems and may present with neurological excitation or depression, hemodynamic instability, and, in severe cases, cardiac arrest. Although uncommon, its clinical significance warrants particular attention given the volumes of local anesthetic frequently used for brachial plexus blockade [95,96,97,98].

**Figure 6:**
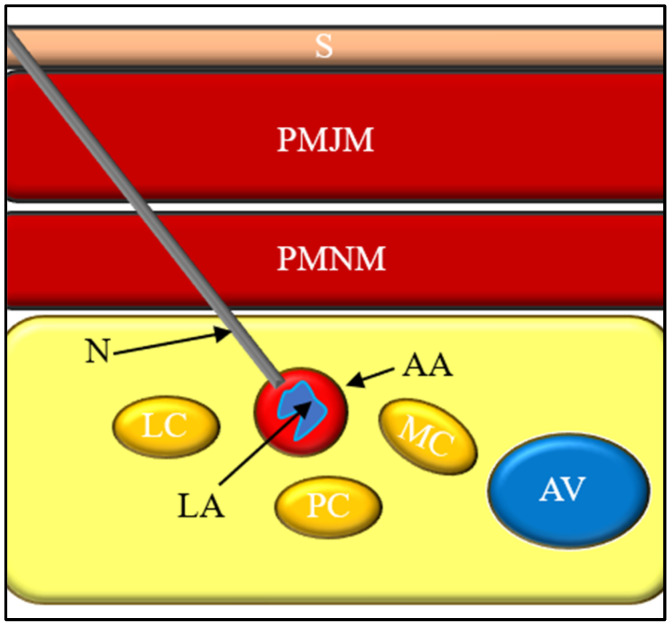
Mechanism of Inadvertent Intra-Arterial Injection During Infraclavicular Brachial Plexus Block.

From a pathophysiological perspective, LAST occurs when plasma concentrations of local anesthetic exceed toxic thresholds. This may result from direct intravascular injection, administration of excessive total doses, or rapid absorption from highly perfused tissues (Figure 6). Early neurological symptoms typically precede cardiovascular toxicity and include perioral numbness, dizziness, tinnitus, dysarthria, altered consciousness, and muscle twitching, followed in more severe cases by seizures or cardiovascular collapse [99,100,101].

Evidence regarding LAST in the context of infraclavicular block is largely derived from pharmacologic principles, registry data, and isolated case reports rather than from large infraclavicular-specific randomized trials.

Clinical studies illustrate both early and delayed manifestations of LAST following infraclavicular block. Yang et al. [36] described two cases of central nervous system toxicity occurring 24–28 min after administration of high doses of ropivacaine without epinephrine, presenting with neurological symptoms and seizures. Both patients recovered following timely supportive treatment [36]. Delayed toxicity has also been reported. İnceöz et al. described a case in which neurological and cardiovascular symptoms developed several hours after block performance, requiring treatment in an intensive care unit [37].

Pharmacological factors play a significant role in determining the risk of toxicity. Ropivacaine and bupivacaine are widely used in infraclavicular block due to their prolonged duration of action; however, both agents have well-described dose-dependent neurotoxic and cardiotoxic profiles. Previous studies indicate that total doses of ropivacaine reaching or exceeding 300 mg may be associated with central nervous system toxicity [102,103,104].

Prevention of LAST is based on a combination of anatomical knowledge, dose optimization, and meticulous technique. Real-time ultrasound guidance, fractionated injection of local anesthetic with intermittent aspiration, avoidance of unnecessarily high volumes, and the use of epinephrine as a marker of intravascular injection may reduce risk. Early recognition of prodromal symptoms and immediate initiation of therapeutic protocols, including lipid emulsion therapy, are essential for minimizing morbidity and mortality.

In summary, although LAST remains a rare complication of infraclavicular brachial plexus block, its potential severity underscores the importance of careful patient selection, appropriate dosing strategies, and continuous vigilance during and after block placement.

#### 3.3.5. Infectious Complications

Infectious complications following infraclavicular brachial plexus block are rare but may be clinically significant when they occur. Reported infections range from localized skin and soft tissue infections at the puncture site to deeper infections associated with the use of perineural catheters for prolonged analgesia. Overall, the incidence of infection after single-shot peripheral nerve blocks is low, whereas catheter-based techniques are associated with a higher, although still limited, risk [105,106,107,108].

The infraclavicular region has specific anatomical and technical characteristics that may influence the risk of infection. The block is typically performed in a relatively deep anatomical plane and may require multiple needle adjustments to achieve optimal distribution of local anesthetic. In addition, the infraclavicular approach is frequently used for prolonged postoperative analgesia with placement of a perineural catheter, which increases the duration of tissue exposure and the potential for microbial colonization [44].

Several patient- and procedure-related factors have been identified as contributing to infection risk. These include inadequate skin antisepsis, multiple needle passes, prolonged catheter dwell time, immunosuppression, diabetes mellitus, and breaches in sterile technique. The most commonly isolated pathogens in infections associated with peripheral nerve blocks are *Staphylococcus aureus* and coagulase-negative staphylococci, reflecting skin flora as the primary source of contamination [44,108,109].

Large cohort studies confirm the low overall incidence of clinically significant infections but demonstrate differences among techniques. In a retrospective analysis of nearly 27,000 brachial plexus blocks, Kubulus et al. found that the infraclavicular approach was associated with a higher rate of catheter-associated infections compared with other brachial plexus block techniques, despite similar block success rates [44,109]. These findings highlight the importance of strict aseptic technique, particularly when infraclavicular catheters are used.

Prevention of infectious complications is based on adherence to established infection control measures, including meticulous skin preparation, use of sterile covers and gel for the ultrasound probe, minimization of needle passes, and careful catheter management. Ultrasound guidance may indirectly reduce infection risk by improving block accuracy and limiting tissue trauma; however, direct comparative data evaluating infection incidence between ultrasound-guided and landmark-based infraclavicular techniques remain limited [110].

In conclusion, infectious complications appear to be uncommon and potentially preventable with strict adherence to aseptic technique. Awareness of patient-related risk factors and meticulous adherence to aseptic technique are essential, particularly when prolonged peripheral nerve blockade is employed.

The included studies and their reported complications are summarized in Table 2.

A considerable proportion of the available evidence on complications of infraclavicular brachial plexus block derives from isolated case reports and small observational studies. While such reports are valuable in identifying rare or unexpected events, they do not permit reliable estimation of true incidence rates or definitive mechanistic conclusions. Mechanistic interpretations based on limited or anecdotal data should therefore be approached with caution. Consequently, interpretations regarding anatomical spread patterns or causality should be regarded as hypothesis-generating rather than confirmatory. Larger prospective studies are required to better define the strength and consistency of these associations.

## 4. Influence of Contemporary Ultrasound-Guided Strategies

### 4.1. Influence of Contemporary Ultrasound-Guided Strategies on Local Anesthetic Spread and Complication Profiles

The increasing use of ultrasound guidance has fundamentally transformed infraclavicular brachial plexus blockade from a largely landmark- or neurostimulation-guided technique into one in which needle trajectory, tip position, and injectate spread can be actively visualized and modified in real time [54,55]. Importantly, ultrasound does not merely “reduce risk”; rather, it enables deliberate manipulation of injectate distribution within the neurovascular compartment and its surrounding tissue planes. As a result, complication profiles may be influenced not only by patient anatomy but also by contemporary procedural choices such as low-volume strategies, needle tip positioning, hydrodissection, and selection of infraclavicular variants (e.g., paracoracoid vs. costoclavicular approaches) [32,40,54,55,85]. The available literature is heterogeneous (including cohorts, small trials, and case reports), and mechanistic interpretations should therefore be considered hypothesis-generating; nevertheless, several clinically relevant themes consistently emerge.

#### Low-Volume Strategies and the Concept of “Sufficient” Spread

Historically, infraclavicular blocks were often performed using relatively large volumes to compensate for uncertainty in needle tip location and injectate distribution. With ultrasound-guided visualization of the cords, axillary vessels, and spread of local anesthetic, there has been a shift toward “minimum effective volume” concepts [54,55,67]. Low-volume strategies may influence complication risk through several mechanisms. First, reduced injectate volume may decrease the likelihood of extensive proximal or medial spread through contiguous tissue planes, which may contribute to unintended sympathetic blockade (e.g., Horner’s syndrome) and phrenic nerve involvement or diaphragmatic dysfunction [40,67]. Second, lower total dose may reduce the probability of systemic toxicity, particularly when combined with fractionated injection and careful aspiration [98,99,101]. Third, smaller volume may lessen compressive effects within relatively constrained tissue planes, potentially reducing injection pressure and discomfort during injection. However, low-volume approaches may be more sensitive to suboptimal needle tip placement, as insufficient distribution around the target cords can result in patchy block or necessitate additional needle passes—an important countervailing consideration, since multiple needle redirections may increase the risk of vascular puncture or inadvertent intraneural needle positioning [38].

### 4.2. Needle Tip Positioning and Pattern of Injectate Distribution

Ultrasound guidance allows the operator to intentionally position the needle tip relative to the axillary artery and the cords (e.g., posterior/lateral versus medial positions), and to observe whether injectate spreads along desired pathways [32,54,55]. Tip position matters because the cords are not uniformly distributed around the artery in all patients and may appear clustered or separated depending on transducer position, arm position, and the infraclavicular variant used. In practice, a key safety objective is maintaining continuous visualization of the needle tip, avoiding “blind advancement,” and ensuring that the needle remains within the intended tissue plane rather than traversing vascular structures or approaching the chest wall [54,55].

From a mechanistic perspective, the infraclavicular neurovascular structures are enclosed within a compartment/tissue plane system that can channel injectate. When local anesthetic is deposited within an appropriate plane adjacent to the cords and vessels, spread may preferentially follow low-resistance paths along connective tissue planes rather than dispersing uniformly [76]. Conversely, if the needle tip is positioned too deep or redirected medially/posteriorly, injectate may track toward the chest wall or pleura-adjacent planes, increasing risk for pleural injury in the setting of excessive advancement or loss of tip visualization [42,43,92,93,94]. Therefore, “needle tip discipline” (continuous visualization, shallow incremental advancement, and confirmation of safe spread before full dosing) represents a central contemporary safety principle and is directly relevant to rare but serious complications such as pneumothorax [54,55,92].

Contemporary ultrasound literature also emphasizes that atypical vascular patterns may be encountered (“vascular signatures”) that warrant heightened caution or modification of the planned needle path, particularly in parasagittal infraclavicular techniques [111]. In cadaveric models, ultrasound-guided single needle tip placement below the axillary artery has been explored as a targeting strategy, reinforcing that small changes in tip position can meaningfully alter perivascular spread patterns and potentially reduce the need for repeated needle repositioning [112].

### 4.3. Hydrodissection as a Safety and Targeting Tool

Hydrodissection—typically performed using small aliquots of saline or dilute local anesthetic—can be used to open tissue planes, improve discrimination between neural and vascular structures, and create a safer working space for subsequent anesthetic deposition. Conceptually, hydrodissection may reduce complications through (i) improved needle tip confirmation (a visible fluid plane forms precisely at the needle tip), (ii) separation of the cords from adjacent vessels in anatomically crowded conditions, and (iii) facilitation of injectate spread within neurovascular tissue planes without the need for aggressive needle repositioning [35,53]. In addition, hydrodissection may help avoid intraneural injection by demonstrating whether fluid dissects around, rather than within, neural structures [58,59,60].

While the available evidence suggests potential benefits, hydrodissection should not be viewed as eliminating risk. It is a technique-dependent adjunct: improper use (e.g., injecting against high resistance, injecting without clear tip visualization, or using excessive volumes) could still contribute to unintended spread or tissue disruption. Accordingly, hydrodissection is best regarded as a contemporary strategy that may improve procedural precision and potentially reduce specific risks (notably vascular puncture in challenging anatomy), rather than as a definitive protective measure [35].

### 4.4. Contemporary Infraclavicular Variants: Paracoracoid, Parasagittal/Vertical, and Costoclavicular Approaches

Multiple infraclavicular variants exist, and the choice of approach can influence both anatomical relationships and complication patterns. Traditional parasagittal or vertical techniques may position the needle trajectory closer to the chest wall depending on the plane and depth, which has been associated in the literature with pleural risk when advancement exceeds the target depth or when the needle is redirected medially or posteriorly [33,42,92]. In contrast, ultrasound-guided paracoracoid approaches often emphasize in-plane visualization with controlled depth, potentially improving safety margins; however, the axillary vessels remain in close proximity and vascular puncture remains possible, particularly with inadequate visualization or rapid needle movement [38].

The costoclavicular approach has gained popularity as an ultrasound-guided variant in which the cords may appear clustered in a more compact arrangement, potentially allowing effective blockade with a more confined injectate distribution [85]. This configuration may theoretically promote more predictable spread within the neurovascular compartment and reduce the need for multiple injections; however, robust comparative data remain limited and reported outcomes vary according to operator experience and technique. Additionally, because the costoclavicular approach is performed in a defined sonographic window beneath the clavicle, needle path and depth control can be optimized; nevertheless, the proximity of vascular structures persists, and meticulous needle tip visualization remains essential. Reports specifically emphasize the importance of careful identification of axillary vessels during ultrasound-guided costoclavicular blockade to reduce vascular complications [113].

Cadaveric studies comparing multiple ultrasound-guided infraclavicular approaches further support that technical variations can materially influence needle trajectory relative to vascular structures and tissue planes [114]. Additional cadaveric data also describe targeted needle-tip strategies (e.g., placement below the axillary artery), supporting the concept that subtle differences in tip position may alter spread patterns within the neurovascular compartment [112].

Overall, contemporary practice increasingly tailors the infraclavicular variant to patient anatomy (body habitus, clavicular/coracoid landmarks, cord visibility, vessel position) and procedural goals (single-injection vs. multi-injection strategies, catheter placement, need for rapid onset vs. safety constraints). These choices plausibly influence complication profiles via differences in needle trajectory relative to the chest wall and via the tissue-plane pathways available for injectate spread [54,55].

### 4.5. Implications for Specific Complication Categories

Vascular puncture and hematoma. Ultrasound guidance facilitates identification of the axillary artery and vein, recognition of anatomic variants, and selection of a needle path that avoids vascular structures [38,54,55]. Low-volume dosing and deliberate needle tip positioning may reduce repeated needle passes, while hydrodissection may aid in separating crowded structures in difficult anatomy [35,67]. However, vascular puncture remains possible, particularly when the needle tip is lost from view or when the operator relies on visualization of the needle shaft rather than direct visualization of the tip [38]. Contemporary reports additionally highlight the importance of recognizing vessel anatomy during costoclavicular blockade and identifying atypical vascular patterns that may warrant modification or avoidance of standard needle paths [111,113].

Neurologic symptoms and nerve injury. Ultrasound may reduce the likelihood of intraneural injection by enabling real-time visualization, but transient paresthesia, dysesthesia, or pain during injection still occur [38,48]. From a mechanistic perspective, risk is influenced by needle tip behavior (contact with neural structures), injection pressure or resistance, and whether injectate dissects around or within neural structures [58,59,60,62,63]. Low-volume approaches may reduce widespread neural exposure but can increase the need for precise pericordal deposition, making technique quality particularly important [67].

Phrenic nerve involvement/diaphragmatic dysfunction. Contemporary low-volume and targeted injection strategies may reduce proximal spread, and manipulation of tissue-plane dynamics (including external supraclavicular pressure in selected contexts) has been proposed as a means to limit cranial migration of injectate. Nevertheless, diaphragmatic dysfunction has been reported following infraclavicular techniques, supporting the concept that tissue-plane continuity can permit proximal spread under certain conditions [39,40,85]. Therefore, the goal in contemporary practice is to reduce risk through volume limitation and careful control of injectate spread, rather than to assume elimination of phrenic involvement [40,41].

Pneumothorax. Modern ultrasound guidance likely reduces pneumothorax risk primarily by improving depth control and enabling continuous needle tip visualization [43]. When pneumothorax occurs, reports frequently implicate loss of needle tip visualization, excessive advancement, or deviation into a deeper plane adjacent to the chest wall [42,43,93,94]. Accordingly, a contemporary emphasis on in-plane approaches, shallow incremental advancement, and repeated confirmation of safe injectate spread before full dosing is central to preventing this rare but serious complication [43,54,55].

Local anesthetic systemic toxicity (LAST). Modern practice often combines ultrasound-guided targeting (potentially reducing required volume), incremental dosing with frequent aspiration, and dose calculations tailored to patient weight and comorbidities [98,99,101]. These measures plausibly reduce the likelihood of intravascular injection and high peak plasma concentrations. Nonetheless, LAST remains possible due to inadvertent intravascular injection, high total dose, or rapid systemic absorption, and vigilance during and after block placement remains essential [36,99,100,101].

### 4.6. Summary

In contemporary practice, the safety profile and spectrum of complications associated with infraclavicular brachial plexus block are shaped not only by regional anatomy but also by modifiable procedural factors. Ultrasound enables active control of needle tip position and injectate spread, while strategies such as low-volume dosing, hydrodissection, and selection among infraclavicular variants may influence tissue-plane dynamics and thereby alter both efficacy and risk [35,40,67,85,111,112,113,114]. Although current evidence is heterogeneous and often non-comparative, these evolving techniques are integral to modern clinical practice and should be considered when interpreting complication mechanisms and developing risk-mitigation strategies [54,55].

## 5. Conclusions

Infraclavicular brachial plexus block is an effective and widely used technique for anesthesia and analgesia in upper limb surgery. Its clinical value is grounded in well-established anatomical principles and the relatively predictable spread of local anesthetic within the brachial plexus. A detailed understanding of the topographical relationships among neural structures, the axillary vessels, and surrounding connective tissue is essential for optimizing both the safety and efficacy of the procedure.

Despite its favorable safety profile, infraclavicular brachial plexus block is not devoid of potential risks. Clinically documented complications—including vascular puncture, nerve injury, Horner’s syndrome, diaphragmatic paralysis, pneumothorax, local anesthetic systemic toxicity and infectious complications—underscore the importance of meticulous technique and routine use of modern guidance modalities such as ultrasound. Accumulating evidence suggests that the incidence of serious adverse events may be substantially reduced through appropriate patient selection, thorough knowledge of anatomical variations, careful needle manipulation, and judicious selection of local anesthetic type, concentration, and volume.

In summary, the infraclavicular block remains a reliable and valuable component of regional anesthesia practice when performed by experienced clinicians with a comprehensive understanding of both its anatomical basis and potential complications. Ongoing research and continued technological advances are expected to further improve the safety, precision, and clinical outcomes associated with this technique.

## Figures and Tables

**Table 1:** Qualitative Comparison of Reported Complications Across Infraclavicular Block Techniques.

Complication Category	Landmark/Vertical Techniques	Nerve Stimulator-Guided	Ultrasound-Guided
Vascular puncture/Hematoma	Frequently reported (e.g., Kilka 1995 [33]; Jandard 2002 [34])	Reported; related to needle proximity without visualization	Reduced incidence but still reported (e.g., Er 2022 [35]); risk persists if needle tip not visualized
Intravascular injection/LAST	Reported, often related to larger volumes and absence of visualization	Reported; aspiration-dependent detection	Reported despite US guidance (Yang 2012 [36]; İnceöz 2015 [37]); reduced risk with incremental injection
Transient neurological symptoms/Paresthesia	Reported, often associated with paresthesia-based placement	Frequently described during motor-response targeting	Lower incidence in large cohorts (Lecours 2013 [38]), but not eliminated
Persistent nerve injury	Rare; described in isolated reports	Rare; primarily transient symptoms	Rare; large series show very low permanent deficit rates
Horner’s syndrome	Reported with larger volumes and vertical approaches	Reported	Reported; likely volume- and spread-dependent rather than technique-specific
Hemidiaphragmatic paralysis	Described (Rettig 2005 [39]—vertical approach)	Reported	Reported; appears volume-dependent (Petrar 2015 [40]; Parameswari 2025 [41])
Pneumothorax	Classically associated with vertical approaches (Neuburger 2000 [42])	Rare but reported	Rare; reported despite US (Gauss 2014 [43]); usually linked to loss of needle tip visualization
Infectious complications	Rare	Rare	Rare; slightly increased with catheter techniques (Kubulus 2024 [44])

**Table 2:** Published studies reporting complications associated with infraclavicular brachial plexus block.

Author	Year	Study Type	No. of Performed Infraclavicular Blocks	Reported Complications
Kilka et al. [33]	1995	Original article	175	Vascular puncture (*n* = 18); Horner’s syndrome (*n* = 12)
Neuburger et al. [42]	2000	Case report	1	Pneumothorax (*n* = 1)
Jandard et al. [34]	2002	Original article	100	Vascular puncture (*n* = 5); Horner’s syndrome (*n* = 4); LAST (*n* = 1)
Gentili et al. [86]	2002	Case report	1	Hemidiaphragmatic paralysis (*n* = 1); Pneumothorax (*n* = 1)
Rodríguez et al. [47]	2004	Original article	60	Vascular puncture (*n* = 1); Hematoma (*n* = 2); Horner’s syndrome (*n* = 1)
Rettig et al. [39]	2005	Original article	35	Horner’s syndrome (*n* = 4); Change in hemidiaphragmatic movement (*n* = 9)
Keschner et al. [64]	2006	Prospective study	248	No complications reported
Salengros et al. [74]	2007	Case report	1	Horner’s syndrome (*n* = 1)
Crews et al. [93]	2007	Case report	1	Pneumothorax (*n* = 1)
Sanchez et al. [94]	2008	Case report	2	Pneumothorax (*n* = 2)
Koscielniak-Nielsen et al. [48]	2009	Original article	60	Vascular puncture (*n* = 1); Paresthesia or pain during injection (*n* = 8)
Fredrickson et al. [65]	2009	Original article	30	Neurological symptoms at day 10 (*n* = 5); Paresthesia during procedure (*n* = 3)
Fredrickson & Kilfoyle [66]	2009	Prospective study	122	Transient neurological symptoms <1 month (*n* = 1); Persistent symptoms 1–6 months (*n* = 8)
Gleeton et al. [49]	2010	Case report	1	Axillary hematoma (*n* = 1)
Yang et al. [50]	2010	Clinical research article	50	Vascular puncture (*n* = 7); Horner’s syndrome (*n* = 4)
Lahori et al. [51]	2011	Clinical investigation	30	Vascular puncture (*n* = 2)
Walid et al. [75]	2012	Case report	1	Horner’s syndrome (*n* = 1)
Yang et al. [36]	2012	Letter to the editor	2	LAST (*n* = 2)
Lecours et al. [38]	2013	Original investigation	627	Upper extremity weakness, pain, or sensory deficits (*n* = 4)
Gauss et al. [43]	2014	Original article	2963	Pneumothorax (*n* = 2)
Petrar et al. [40]	2015	Original article	32	Complete hemidiaphragmatic paralysis (*n* = 1); Partial or complete paralysis (*n* = 4); Dyspnea (*n* = 5)
İnceöz et al. [37]	2015	Case report	1	LAST (*n* = 1)
Vazin et al. [67]	2016	Clinical study	40	Late dysesthesia potentially related to nerve block (*n* = 5)
Abhinaya et al. [52]	2017	Original article	30	Vascular puncture (*n* = 1)
Er et al. [35]	2022	Clinical trial	85	Arterial puncture (*n* = 4)
Parameswari et al. [41]	2025	Original article	30	Partial hemidiaphragmatic paralysis (*n* = 1); Accidental vascular puncture (*n* = 1)

## Data Availability

No new data were created or analyzed in this study.
